# Revie ⊕: the influence of a life review intervention including a positive, patient-centered approach towards enhancing the personal dignity of patients with advanced cancer—a study protocol for a feasibility study using a mixed method investigation

**DOI:** 10.1186/s40814-016-0101-z

**Published:** 2016-10-19

**Authors:** Maria Goreti Da Rocha Rodrigues, Sophie Pautex, Maya Shaha

**Affiliations:** 1 Institute of Higher Education and Research in Healthcare, University of Lausanne and University Hospital of Lausanne, 10 Route de la Corniche, 1010 Lausanne, Switzerland; 2 School of Health Sciences, University of Applied Sciences and Arts Western Switzerland, 47 Avenue de Champel, 1206 Genève, Switzerland; 3 Department of Community Medicine, Geneva University Hospitals and Geneva University, 4 rue Gabrielle-Perret-Gentil, Genève, 14 1211 Switzerland; 4 Inselspital, Bern University Hospital, Freiburgstrasse, 3010 Bern, Switzerland

**Keywords:** Advanced cancer, Palliative care, Life review intervention, Posttraumatic growth, Dignity, Satisfaction with life, Mixed methods

## Abstract

**Background:**

It is generally recognized that existential concerns must be addressed to promote the dignity of patients with advanced cancer. A number of interventions have been developed in this regard, such as dignity therapy and other life review interventions (LRI). However, so far, none have focused on a positive approach or evaluated its effects on dignity and personal growth. This study aims to explore the feasibility of Revie ⊕, a life review intervention comprising a positive, patient-centered approach, and to determine potential changes of patients’ sense of dignity, posttraumatic growth, and satisfaction with life.

**Methods:**

A mixed method study will be performed, which includes specialized nurses and 40 patients with advanced cancer in an ambulatory and in-patient setting of a Swiss university hospital. Quantitative methods involve a single group, pre- and post-intervention, and outcome measurements include the Patient Dignity Inventory, the Posttraumatic Growth Inventory, and the Satisfaction with Life Scale. Feasibility data relating to process, resource, and scientific elements of the trial will also be collected. A semi-directed interview will be used to collect qualitative data about the process and the participants’ experiences of the intervention. In this way, enhanced quantitative-qualitative evidence can be drawn from outcome measures as well as individual, contextualized personal views, to help inform researchers about the plausibility of this complex intervention before testing its effectiveness in a subsequent full trial.

**Discussion:**

Patient dignity is a goal of quality end-of-life care. To our knowledge, this is the first trial to evaluate the role of a life review intervention that is focused on personal growth and on changes relating to the experience of having cancer.

This study will evaluate the feasibility of a novel intervention, Revie ⊕, which we hope will contribute to promote the dignity, personal growth, and overall life satisfaction of patients with advanced cancer.

**Trial registration:**

ISRCTN, ISRCTN12497093

**Electronic supplementary material:**

The online version of this article (doi:10.1186/s40814-016-0101-z) contains supplementary material, which is available to authorized users.

## Background

Approximately 37,000 new cases of cancer occur annually in Switzerland. Despite advancements in medical technology, cancer remains the second leading cause of mortality (about 16,000 individuals per year) in Switzerland [1]. Patients with advanced cancer often experience significant levels of existential distress [2, 3]. According to Kissane [4], existential distress represents a person’s distress when facing his/her own mortality, which brings out feelings of helplessness, lack of sense, discouragement, or remorse. Existential suffering is one of the most exhausting conditions at the end of life [5]. There is a paucity of interventions focusing on relieving spiritual or existential concerns [2]. However, it is recommended to consider the patients’ and families’ values and beliefs; to help patients and families in sharing their emotions and time; and to support patients and families in carrying out realistic projects [6, 7]. Interventions that relieve existential distress support patients’ dying with dignity [8, 9]. In end-of-life care, dying with dignity constitutes an important aim, but this concept has multiple meanings. According to Guo and Jacelon [10], dying with dignity includes a dying process with minimal symptom distress, being human and being self, achieving existential and spiritual goals, and maintaining meaningful relationships with significant relatives*.*

Dignity at the end of life is a major concern of health professionals in care of people with advanced cancer [11]. Nurses are most in contact with patients facing life-threatening disease and they play a specific role in providing quality of life, supporting health and well-being [12, 13].

Despite continued developments and improvements in palliative care in Switzerland, nursing interventions promoting patient dignity are still insufficiently available in routine clinical practice. To date, little empirical research has focused on interventions to address existential distress [2]. One way of addressing these needs and enhancing dignity during the end of life is a therapeutic life review intervention (LRI) [14]. This set of interventions consists of evoking memories, personal life events, and accomplishments. It is a structured process of reinterpreting life existence [15].

LRIs in palliative situations have been shown to have positive effects on quality of life [16, 17], spiritual well-being [18], depression [19, 20], and death-related anxiety [21]. The main perceptions of patients are reconsideration and acceptance of the uniqueness of their lives, finding meaning, defining future projects, reconciliation with their past experiences [22], and the satisfaction of leaving a personal legacy [23]. Change also occurs within the family in terms of perception, satisfaction [23–25], communication, and cohesion [26, 27].

Despite cancer being widely seen as an adverse event, some cancer patients report having experienced a degree of personal growth that caused them to change their values and relationships in positive ways. Tedeschi and Calhoun [28, 29] defined this phenomenon as posttraumatic growth (PTG). It refers to the positive psychological change experienced as a result of struggles with highly challenging life circumstances. In the context of experiencing advanced cancer, PTG is documented. Changes can occur through the intensification of human relationships, a better outlook on life [30], greater religious satisfaction, increased compassion, and concern for others [31]. The main positive outcomes of PTG are decreased psychological distress [32] and depression [33, 34], as well as enhanced levels of general [31, 35] and spiritual well-being [33].

While some LRIs have already been shown to have a benefit at an existential level among individuals with advanced cancer, none, to our knowledge, are relying on an approach centered on personal development, although it has been shown that PTG can have a positive influence on the well-being of patients with advanced cancer [8]. Moreover, this type of intervention has not yet been tested in the context of specific conditions and circumstances that may be encountered in Switzerland.

In our current research, we propose to develop a specific LRI to promote existential needs in advanced cancer patients. We used the MRC framework for the development of complex interventions [36] with the aim to build on the World Health Organization’s (WHO) recommendations for palliative care that emphasize the importance of spiritual well-being. The intervention is called Revie ⊕. It focuses on personal resources as well as changes in relationships and values since the time of diagnosis. The intervention is patient-centered and consistent with the patient’s needs, values, and preferences. Orienting the intervention to include a positive approach, namely to expand on the positive changes brought on by the cancer experience, could contribute to promote dignity, personal development, and a higher degree of overall life satisfaction.

The purpose of the present study is to assess the feasibility of the Revie ⊕ intervention and to evaluate potential changes in dignity, posttraumatic growth, and satisfaction of life for patients with advanced cancer in order to obtain data for a future effectiveness trial.

## Methods/design

### Aim/objectives

The overall aim of this study is to evaluate the feasibility of Revie ⊕, a new LRI intervention aimed at patients with advanced cancer within the ambulatory and in-patient setting of a university hospital in the French-speaking part of Switzerland, and to evaluate potential changes in dignity, posttraumatic growth, and satisfaction with life.

Our primary objectives relate to the assessment of the feasibility of the proposed recruitment strategies and outcome assessment protocols:

1. Determine recruitment and retention rates and identify best strategies for recruitment and retention in this patient population (if 40 participants are recruited over 12 months and with 80 % having completed follow-up, the intervention is considered feasible).
2. Determine the acceptability of the intervention for the patients, in terms of:
  - Engagement and compliance with the protocol
  - Perceptions of barriers and facilitators considering the sensitive and intimate topic and the particular vulnerability of the population with life-threatening disease
  - The degree of satisfaction
  - The perceived relevance of the intervention
3. Explore the acceptability for nurses delivering the intervention in terms of fidelity (adherence structured content), resources mobilized, perception of barriers, and facilitators, considering the sensitive and intimate discussion and practice change.
4. Estimate required sample size for a future larger effectiveness trial.

The secondary objectives will be to assess the impact of Revie ⊕ on the sense of dignity, posttraumatic growth, and satisfaction with life for patients with advanced cancer and to determine the most appropriate outcome for a future larger trial.

### Study design

We propose a pilot pre-post feasibility study using a mixed method approach, i.e., an embedded concurrent design with both quantitative and qualitative parts [37].

The quantitative part of the study consists of a single pre-post-intervention group. The qualitative method is nested within the predominant quantitative part and includes semi-directed interviews with the patients to obtain data on the intervention process and participants’ experiences and views.

Questionnaires and a focus group interview are used to explore the health professionals’ acceptability.

An embedded concurrent design has been chosen because the combination of quantitative and qualitative methods will provide more information about the barriers for participation, and an estimated response rate. This study will also help us to better understand the impact of the intervention on the participants (willingness, unanticipated experiences during the trial, resources that can facilitate to conduct the intervention, the feedback of the patients and nurses about the intervention delivery). The use of a mixed method is recommended to improve interpretation of the results in palliative care research [38, 39]. RCT may not be the most appropriate design because of practical and ethical obstacles. At this stage, we did not opt for a control group as we did not intend to measure effectiveness in the first place. Our main purpose was to develop a theory-based intervention, determine the patients’ perception and satisfaction with the intervention, explore the patients’ adhesion to the intervention, determine the appropriateness of the outcome measures, and identify the adequacy of time, location, and process. In addition, it was important to explore the emotions associated with this intervention through the discussion of this intimate and sensitive topic.

### Study setting

A purposive sample of 40 patients with advanced cancer will be recruited within the ambulatory and in-patient setting of a university hospital in the French-speaking part of Switzerland. As it is a pilot study, and the intervention has not been tested previously, no formal sample size calculation was conducted [40]. The sample size was based on the recommendations by Hertzog [41]: a sample of 20–40 persons is adequate for a pilot study employing a single group to ultimately estimate the sample size for a future trial.

This preliminary study reviews the recruitment; checks the feasibility and the appropriateness of the instruments used and the sample size required for a subsequent larger study; and examines how the process (timing, duration, etc.) could eventually be consolidated [36, 42]. Difficulties of recruitment and retention are reported by similar studies, particularly concerning advanced cancer patients and studies over 12 months. However, considering that approximately 800 persons consult annually in the two participating settings and that 40 % of these persons are potentially eligible for the study and estimating an 80 % refusal, it seems feasible to recruit a total of 40 participants.

- *Inclusion criteria*: adults (aged 18 years or older) with advanced cancer (T3 or T4, or the presence of metastases), with an adequate health status to participate in the study, as determined by clinical consensus between nurses and physicians, and who are able to cognitively understand and consent to inclusion in the study
- *Exclusion criteria*: patients diagnosed with cognitive disorders related to memory loss or disturbances of speech that would not allow for a constructive exchange, and people with insufficient command of the French language to complete the study questionnaires

### Recruitment

Recruitment began in April 2015 and will take place over a 12-month period. Information was given prior to the commencement of the study to all the nurses, head nurses, and oncologists of the services. A factsheet was also made available to them. They all have a role to play in informing patients about the study. Eligible patients are invited by the nursing staff to participate in the study. They are subsequently contacted by the researcher(s), who provides an overview of the study and presents the information letter and consent form to the potential participant. A minimum of 24 h is given to the participant to sign the consent form.

Regular meetings are planned between the research team and the staff to identify and overcome any difficulties encountered during this phase to enhance recruitment. To date, 30 participants have been recruited.

### Study overview

The study consists of three stages (Table 1): the pre-intervention phase (T0), followed by the intervention (T1), and, after its completion, the post-intervention phase (T2). In light of recommendations by other investigators who have implemented a similar intervention focused on life review [16, 17], the time between T0 and T2 will be limited to a maximum of 1 month. This timeframe takes into account the rate of potential loss of participants due to their lower general health status or as a result of patient death, and the fact that the effects on the sense of dignity, life satisfaction, and posttraumatic growth appear to occur fairly soon after the intervention.

**Table 1:** Study procedure

T0: pre-test	T1: intervention		T2: post-test	
Quantitative	Session 1	Session 2	Quantitative	Qualitative
Sociodemographic + health data PDI^a^, SWLS^b^, PTGI^c^	Encounter with trained nurse to share life history + positive approach	Presenting and finalizing the booklet	Acceptability questionnaire PDP, SWLS, PTGI	Semi-structured interview about the process

*PDI* Personal Dignity Inventory, *SWLS* Satisfaction with Life Scale, *PTGI* Posttraumatic Growth Inventory

^a^Chochinov et al. [43]

^b^Diener et al. [50]

^c^Tedeschi and Calhoun [46]

### Description of the intervention

A brief life review intervention (Revie ⊕) was developed based on a literature review and a theoretical model focusing on a positive patient-centered approach. Revie ⊕ allows specialist nurses to talk about life events with cancer patients, explore how the diagnosis has changed their values and preferences, and discuss ultimate life goals or projects. As an outcome of this LRI, a booklet will be created by integrating the patient’s significant elements evoked during the intervention. The intervention is delivered by eight nurses, all of whom are certified in oncology care, have received specific theoretical and evidence-based knowledge, and have the specific skills needed to deliver a life review intervention. These nurses are part of the staff. However, these nurses have time to deliver the intervention. The intervention consists of two sessions scheduled in mutual agreement between nurse and patient, but above all favoring an appropriate time for the patient. The face-to-face interview constitutes a moment completely centered on the patient’s experience. This face-to-face interview takes place in a space reserved for this purpose. In order to conduct the interview, the nurses follow a predefined guideline that permits them to be in authentic presence with the participants. The first session (T1) lasts for approximately 60 min and allows participants to share significant events about their life history as well as supports personal development by focusing on positive changes that have occurred since the cancer diagnosis. Five domains are addressed during this session: (1) a reflection of the patient’s life story and specific significant events; (2) a focus on the positive changes that have occurred since the diagnosis; (3) an expression of the patient’s values and vision of life (accentuating the strengths and resources) and the patient’s relationship with others; (4) a discussion of significant issues (i.e., what patients want to communicate with their relatives, or determining the project, which is most important to carry out); and (5) a discussion of the patient’s deepest concerns and his/her thoughts about death and dying. Throughout the intervention, nurses value patients’ coping strategies, strengths, and resources. The changes brought about by the patients’ experience of cancer on themselves and their life vision and values as well as their relationship with others are at the center of the intervention.

At the end of the interview, the participant is asked to send pictures, poems, or drawings that he considers important to the researcher. The tape recordings of the session are transcribed by the research team. Thus, a detailed account of the patients’ life events and experiences are obtained. Based on these transcripts, a booklet is created by the research team. The booklet consists of about 12 pages in A5 format and covers four sections: (1) my significant life events and the significant persons in my life; (2) my experience of cancer; (3) my values/life vision/changes; and (4) my project(s). Each booklet is personalized by highlighting its unique style with chosen words (we strive to be as close as possible to patients’ words and expressions). According to the patients’ wishes, texts, poems, pictures, or citations are integrated into the booklet.

The second meeting (15–30 min duration) is arranged to present, complete, if necessary change, and finalize the booklet. Patients who did not send photos, poems, or any other text before can include these at this moment. Patients are fully integrated as main actors of this process. If needed, another meeting is scheduled to revise the booklet. The final version of the booklet is validated by the patient before printing and delivering.

### Data collection

A baseline assessment will take place at (T0), including collection of the participants’ demographic data and data on their religious/spiritual background, as well as medical data such as tumor type, time since diagnosis, and previous treatments.

### Outcomes measures

The outcomes measures will be collected during two stages (i.e., the pre-intervention T0 and the post-intervention T2).

#### Dignity

The Patient Dignity Inventory (PDI) questionnaire [43] was designed to assess dignity-related distress. It consists of 25 items in three subcategories: illness-related concerns, a dignity conserving repertoire, and a social dignity inventory. The items are rated on a five-point Likert scale ranging from 1 (“not a problem”) to 5 (“an overwhelming problem”). The PDI exhibits good reliability and validity (Cronbach’s coefficient alpha = 0.93), and the test-retest reliability was also good (*r* = 0.85) [44]. A French version has been translated by Gagnon [18] and validated by Chochinov and his team [45].

#### Post-traumatic growth

The Posttraumatic Growth Inventory (PTGI) [46] will be used in our study to describe the change experienced by the patient since his or her cancer diagnosis. It consists of 21 declarative statements, with responses ranking from 0 to 5 (0 = “I did not experience this change”; 5 = “I experienced this change to a great extent”). The PTGI determines five factors: appreciation of life, relating to others, personal strengths, new possibilities, and spiritual change. The PTGI exhibits good internal consistency (Cronbach’s alpha coefficient = 0.90), and an acceptable test-retest reliability (*r* = 0.71) [47]. This instrument has been translated and validated into French (Cronbach’s alpha coefficient = 0.88 and the reliability = 0.47 for the French version) [48, 49].

#### Satisfaction with life

The Satisfaction with Life Scale (SWLS) [50] is used to evaluate patients’ satisfaction with life as a whole. It assesses a given individual’s global assessment of life satisfaction, and hence allows respondents to weight domains of their lives in terms of their own values. The SWLS contains five items and uses a scale of 1–7 (1 = “strongly disagree” and 7 = “strongly agree”). Cronbach’s alpha coefficient is between 0.79 and 0.89, and reliability is *r* = 0.84 [51]. The instrument has been translated into French and Cronbach’s alpha coefficient is between 0.80 and 0.84 for the French version [52]. Reliability and validity indices were validated and compared with those of the original English version.

#### Acceptability questionnaires

The acceptability questionnaire was developed by the research team based on the literature and will be addressed to participants at T2. It uses a scale of 1–5 (1 = “strongly disagree” and 5 = “strongly agree”) to assess five items: (1) the topics discussed during the interview (facility of understanding, if the questions are intrusive, distressing, relevant), (2) the planning of the meetings (the number and spacing), (3) the environment of the intervention (comfort and appropriateness), (4) the booklet (content and presentation), and (5) more generally, whether the intervention is helpful, the satisfaction of the intervention and if the participant would recommend the Revie ⊕ to other patients.

At the end of the study, an acceptability questionnaire addressed to the nurses delivering the intervention will be administrated. The questionnaire was developed by the research team based on the literature. It uses a scale of 1–5 (1 = “strongly disagree” and 5 = “strongly agree”) to assess the same five items as the participants as well as the monitoring protocol, and the training, supervision and debriefing.

### Intervention fidelity

To obtain fidelity, a guide for conducting the intervention was created. The nurses keep the guide during the intervention. During training, the importance of adhering to this guide was emphasized. The interviews are tape-recorded. Subsequently, the research team listens to the tapes to create the booklet. If case deviations are perceived, the researcher asks the nurses to adjust their intervention delivery. Regular meetings between researcher and nurses are planned in which nurses can also express their feelings and views. Finally, the nurses keep a personal diary and can add comments regarding difficulties or barriers to maintain intervention fidelity. At the end of the empirical phase, the diaries will be analyzed.

### Qualitative part

At the end of the intervention and after completing the post-test questionnaires (T2), a semi-structured interview will be conducted by the researcher, which lasts for about 30 min. This interview focuses on the experience of the intervention and the process.

The following questions are addressed: “You shared your life perspectives and finalized a booklet. What do you think and feel about the whole experience and the process? What feelings did you experience most intensely during the process?”

The nurses are also invited to keep a notebook in which they can note their difficulties and their emotions. These writings will be transcribed and a thematic analysis will be performed. To gather more detail about their intervention experience, a focus group will be led by two facilitators who are external to the research.

### Data analysis

The protocol is drafted in accordance with the SPIRIT 2013 statement [53].

#### Quantitative part

Data analysis will be performed using Stata Software Stata©. Descriptive statistics (mean, median, standard deviation) will be calculated for sociodemographic data. Analyses will be carried out on all available patient data with data at baseline and at the end of the study intervention. We will report descriptive findings concerning recruitment numbers, completions, drop-out rates, and missing data. Quantitative data will be cleaned, i.e., checked for out of range values and missing values. For >10 % of missing values, the subject will not be considered. Distribution of variables will be checked for normality using box plot, skewness and kurtosis, and test of normality. The data will be summarized with standard descriptive measurements. Continuous variables will be summarized by their mean and standard deviation, categorical variables as numbers and percentages, and presented with 95 % confidence intervals.

#### Qualitative data

Qualitative data will be managed using MAXQDA software.

The interviews will be transcribed and an inductive thematic analysis will be performed [54]. At least two researchers will independently code the interviews [55]. Reliability will be analyzed by comparing coding among coders, and an intercoder agreement will be checked within the research team. The codes will be assigned into themes.

Using an embedded concurrent design allows qualitative data to complement quantitative data, thus giving a more comprehensive perspective on the phenomenon of the study [37]. This study will also provide data to contribute to sample size calculations for a future larger effectiveness trial.

### Ethics, consent, and permissions

Our study protocol was approved by the Cantonal Commission of Ethics of Scientific Research (reference no. 15-037; see Additional files 1, 2, and 3). All patients in the study will provide written informed consent for study inclusion and the publication of data (albeit anonymously). Given the sensitivity of the topic investigated, there is a minimal risk of disrupting the psychological and emotional comfort of the participant. Nurses who conduct the intervention have professional experience and are specialized in oncology or palliative care. This enables them to provide psychological support during and after the intervention. In the case of severe emotional reaction, an internal resource for psychological support will be offered.

### Data management

The information collected will be treated anonymously and confidentially. All collected information will be encrypted through an identification number (the participant’s name will not appear). Data will be stored in a computer database to which access will be protected by passwords known only to the investigators. Personal data can be made available confidentially for audit or inspection by authorities, and in order to verify the application of ethical principles of good clinical practice in the context of a research protocol. After the transmission of data and analysis, it will become impossible to identify patients in any published articles. All documents related to the research will be archived for 10 years.

Measures to protect the confidentiality of the research data (i.e., privacy, coding, and storage) are included in this study, and as the risk level for participants is low, a data monitoring committee (DMC) was not deemed necessary for this feasibility study.

## Discussion

This new LRI intervention, called Revie ⊕, intends to promote the dignity of patients with advanced stage cancer. This study is innovative because there is a paucity of knowledge and information regarding life review interventions focusing on a positive, person-centered approach in palliative care and how this may apply under current cancer care settings in Switzerland. Life review interventions have already proven to be effective in other countries, such as Canada, Australia, and Asia. It can be expected, therefore, that this study will provide new data that may be of specific relevance to the setting in Switzerland.

This preliminary study should inform about the recruitment process and the adherence and retention of this particularly vulnerable population. It aims to give indications about the feasibility of this innovative intervention and, eventually, how to adapt the content and duration of the intervention. We will obtain information about the helpfulness of the approach by patient questionnaires and interviews. In addition, the perspective of the nurses about the acceptability of this intervention will be elicited. Nurses will be asked to provide their views about this intimate and sensitive intervention.

The use of an embedded design is recommended for testing complex patient-centered interventions, particularly for pilot studies in which new areas are investigated. We will obtain information about the sampling, the intervention delivery, study implementation, and study outcomes and measures [56].

The data will help us to validate the instruments used and determine the required sample size. If the findings from our pilot study suggest that the trial is feasible, a future larger study will be planned to measure the effectiveness of this complex intervention. This study aims also to measure strength and promote patient dignity, rather than focusing on the problems or difficulties that patients may face. Steering the intervention towards a positive approach, that is to say the positive changes that can occur as the direct result of experiencing a cancer diagnosis, could contribute to personal development and a better overall level of satisfaction with life. Being more in tune with the values and preferences of afflicted individuals could substantially contribute to their well-being in the final stages of their lives.

### Study status

The study began in April 2015. To date, 30 participants have been recruited and 27 interventions have been performed (three interventions have yet to be delivered).

## Additional files

Additional file 1: Participant information letter and consent form. (DOC 86 kb)
Additional file 2: SPIRIT 2013 statement checklist: recommended items to address in a clinical trial protocol and related documents. (DOC 123 kb)
Additional file 3: Funding obtained. (PDF 89 kb)

## Abbreviations

LRI: Life review intervention
PDI: Personal Dignity Inventory
PTG: Posttraumatic growth
PTGI: Posttraumatic Growth Inventory
SWLS: Satisfaction with Life Scale
WHO: World Health Organization
