# Supplementary material for: Revie ⊕: the influence of a life review intervention including a positive, patient-centered approach towards enhancing the personal dignity of patients with advanced cancer—a study protocol for a feasibility study using a mixed method investigation
Source: Pilot Feasibility Stud. 2016 Oct 19;2:63. doi: 10.1186/s40814-016-0101-z (PMC5154126; doi:10.1186/s40814-016-0101-z)
Supplement: Additional file 1: — Participant information letter and consent form. (DOC 86 kb) [file 40814_2016_101_MOESM1_ESM.doc]

**Revie ⊕:** **Etude qui vise à promouvoir la dignité auprès de personnes avec un cancer avancé**

**__________________________________________________________________**

**Formulaire de consentement du patient**

**Collaboratrices scientifiques :** Maria Goreti da Rocha Rodrigues (doctorante, Université de Lausanne), sous la co-direction de Docteure Maya Shaha et Docteure Sophie Pautex

Nom:

Prénom:

Date de naissance:

Numéro d’identification du patient:

- Je déclare avoir été informé-e oralement et par écrit des objectifs et du déroulement de l’étude par le collaborateur scientifique
- Je certifie avoir lu et compris l’information écrite aux patients qui m’a été remise sur l’étude précitée. Je comprends la nature et les avantages de ma participation à l’étude ainsi que les inconvénients. J’ai reçu des réponses satisfaisantes aux questions que j’ai posées en relation avec ma participation à cette étude. Je conserve l’information écrite aux patients et reçois une copie de ma déclaration écrite de consentement.
- J’ai eu suffisamment de temps pour prendre ma décision.
- Je sais que mes données personnelles ne seront transmises que sous une forme anonyme à des institutions externes, à des fins de recherche. J’accepte que les collaboratrices scientifiques, la directrice de l’étude et les autorités de la Commission d’éthique cantonale puissent consulter mes données brutes afin de procéder à des examens et à des contrôles, à condition toutefois que leur confidentialité soit strictement assurée. Tous les documents en lien avec la recherche seront archivés pendant 10 ans.
- Je prends part de façon volontaire à cette étude. Je peux, à tout moment et sans avoir à fournir de justification, révoquer mon consentement à participer à cette étude, sans pour cela en subir quelque inconvénient que ce soit dans mon suivi médical ultérieur.
- Je consens par ma signature que je suis d’accord de participer à cette étude.

Lieu et date: ______________________ Lieu et date:_______________________

Signature de la chercheuse: Signature du Patient:

_________________________________ _________________________________
