# Supplementary material for: Revie ⊕: the influence of a life review intervention including a positive, patient-centered approach towards enhancing the personal dignity of patients with advanced cancer—a study protocol for a feasibility study using a mixed method investigation
Source: Pilot Feasibility Stud. 2016 Oct 19;2:63. doi: 10.1186/s40814-016-0101-z (PMC5154126; doi:10.1186/s40814-016-0101-z)
Supplement: Additional file 3: — Funding obtained. (PDF 89 kb) [file 40814_2016_101_MOESM3_ESM.pdf]

PROMESSE DE SUBVENTION RA&D
ENVELOPPE BUDGETAIRE : FRI 2015

Delémont, le 12 février 2016

DOMAINE	Santé
PROGRAMME	Projets de recherche (esquisses, projets internes et compléments financiers)
Référence HES-SO :	56897/S-RAD15-50

REQUERANT-E PRINCIPAL-E : G70

Haute école de santé - Genève
HEdS-GE
Avenue de Champel 47
1206 Genève
A l'att. de Maria Goreti Da Rocha Rodrigues

LIBELLE ET ACRONYME DU PROJET

Étude de faisabilité et acceptabilité de l'intervention Revie (+) autour du récit de vie auprès de personnes avec un cancer avancé - Revie (+)_forfait

MONTANT TOTAL DE LA SUBVENTION : CHF **11'000.00**

COMMUNAUTE DE RECHERCHE		Code école	CHF
- Requérant principal :	HEdS-GE	G70	11'000.00
- Co-requérant n° 1 :	-	-	0.00
- Co-requérant n° 2 :	-	-	0.00
- Co-requérant n° 3 :	-	-	0.00
- Co-requérant n° 4 :	-	-	0.00
- Co-requérant n° 5 :	-	-	0.00
- Co-requérant n° 6 :	-	-	0.00

REFERENCES :
- Demande de subvention du 09.10.2015 Acceptée le 11.12.2015
- R2015/19/53 du 11 mai 2015

CONDITIONS FINANCIERES :

- La présente subvention constitue une limite-plafond, dans le cadre de laquelle le décompte final des coûts subventionnables est déterminant.

- Les versements sont effectués conformément aux présentes règles :

Modalité 2a : Soutien au montage de projets européens ou déposés dans des instances tierces

pour un montant relatif au programme soutien au montage de projets européens ou déposés dans des instances tierces : VERSEMENT UNIQUE

Le versement unique est effectué par les services centraux de la HES-SO sur présentation d'une facture avec BV-BVR, avec la mention "demande de transfert de subvention » et les références de la présente promesse.
Attention, le financement du montage doit être renseigné dans un compte SageX réservé exclusivement aux montages de projets (1 compte SageX par année budgétaire). Ce compte SageX devra être indépendant du projet SageX financé par l'Union européenne ou par l'instance tierce. Ce compte SageX fera l'objet d'un chapitre au rapport d'activités annuel du domaine. L'obligation de saisir les heures dans SageX n'est pas nécessaire.

Philippe Longchamp
P. Longchamp
Coordinateur de la commission scientifique du domaine Santé

Annabelle Sanchez
Gestionnaire financière des fonds

COPIES :
- à la direction des écoles du/de la requérant-e principal-e et des co-requérant-e-s
- au/à la responsable du programme
- aux responsables financiers des établissements du/de la requérant-e principal-e et des co-requérant-e-s
